# Effects of the methacrylate/acrylate monomers HEMA, TEGDMA, DEGDA, and EMA on the immune system

**DOI:** 10.1002/cre2.93

**Published:** 2017-11-17

**Authors:** Sara Alizadehgharib, Anna‐Karin Östberg, Ulf Dahlgren

**Affiliations:** ^1^ Department of Oral Microbiology and Immunology University of Gothenburg, The Sahlgrenska Academy, Institute of Odontology PO Box 450, SE‐405 30 Gothenburg Sweden

**Keywords:** acrylate, cytokines, dental materials, dentin‐bonding agents, immunoglobulin G, methacrylate

## Abstract

Incomplete curing of dental fillings may lead to leakage of methacrylate/acrylate monomers, which may come in contact with different cells of the immune system in oral tissues. Very little is known about the different immunologic effects caused by these methacrylates/acrylates. The objective of the present study was to study if and how the methacrylate/acrylate monomers ethyl methacrylate (EMA) and diethylene glycol diacrylate (DEGDA) affect the immune system in vivo and in vitro in comparison to 2‐hydroxyethyl methacrylate (HEMA) and triethylene glycol dimethacrylate (TEGDMA). Human peripheral blood mononuclear cells were exposed to the different monomers (500 and 1000 μM) for 24 hr in vitro. BioPlex Pro™ assays were used for cytokine analysis. In vivo, BALB/c mice were immunized subcutaneously at the base of the tail with HEMA, TEGDMA, EMA, or DEGDA in combination with ovalbumin (OVA) in order to study adjuvant properties of the 4 monomers. Peripheral blood mononuclear cells exposed to DEGDA had viability less than 50% of the cells. A pattern was observed where the levels of most cytokines were elevated after exposure to HEMA or TEGDMA. Since that, many cells died after DEGDA‐exposure, the only observed cytokine secretion was a significantly increased production of interleukin‐18. In the in vivo experiments, all mice immunized with DEGDA died after the booster injection. Mice receiving OVA in combination with HEMA, TEGDMA, or EMA developed a higher immunoglobulin G anti‐OVA antibody levels compared to the group immunized with OVA alone. We could not demonstrate any significant difference in antibody levels among the mice receiving the various methacrylate/acrylate monomers. The different monomers affected the production, increase and decrease, of different cytokines in vitro but resulted also in vivo in increased antibody production and T‐cell activity.

## 1 INTRODUCTION

Dental resin‐based composites consist of an organic polymerizable matrix, an inorganic reinforcing filler and a coupling agent that interconnects the organic and inorganic matrixes. The organic polymerizable matrix consists of methacrylate/acrylate monomers and various additives (e.g., initiator, co‐initiator, inhibitor of polymerization, and a photostabilizer; Geurtsen, 1998). The most commonly used monomers in dental materials include 2‐hydroxyethyl methacrylate (HEMA) and triethylene glycol dimethacrylate (TEGDMA; Estlander & Jolanki, 2006). Two other regularly used methacrylate/acrylate monomers are ethyl methacrylate (EMA) and diethylene glycol diacrylate (DEGDA). Previous studies have shown that these compounds frequently trigger contact dermatitis in dental personnel (Aalto‐Korte, Alanko, Kuuliala, & Jolanki, 2007). Uncured residual monomers leak from newly installed composite fillings and may reach the oral mucosa and the dental pulp (Noda et al., 2002; Van Landuyt et al., 2011), where they come in contact with different cells of the immune system. In addition, methacrylate/acrylate monomers may appear in the oral cavity due to wear and/or erosion of composite materials (Geurtsen, 1998).

We have previously shown that methacrylate monomers penetrate intact skin and induce immunologic reactions in draining lymph nodes (Sandberg & Dahlgren, 2006). During a response in a lymph node, many different cytokines are produced, some of which are pro‐inflammatory (e.g., interleukin [IL]‐1, IL‐6, and tumor necrosis factor [TNF]‐α) and some of which are anti‐inflammatory (IL‐10 and transforming growth factor‐ß). Furthermore, chemokines, which are small chemotactic cytokines, are produced that lead to the recruitment of leukocytes from the blood.

Previous studies have shown that the methacrylate monomers HEMA and TEGDMA have multiple effects on the immune system (Andersson & Dahlgren, 2010, 2011a, 2011b; Sandberg, Bergenholtz, Kahu, & Dahlgren, 2005; Sandberg & Dahlgren, 2006; Sandberg, Kahu, & Dahlgren, 2005). Among the effects demonstrated are reactive oxygen species formation, concentration‐dependent apoptosis, phosphorylation of extracellular signal‐regulated kinase (ERK) (Samuelsen, Dahl, Karlsson, Morisbak, & Becher, 2007), and attenuation of Lipopolysaccharides (LPS)‐induced cytokine release from the macrophage cell line RAW264.7 (Bolling et al., 2013). Other studies have shown that HEMA and TEGDMA cause the production of IL‐1β and monocyte chemotactic protein‐1 in vitro (Gregson, Terrence O'Neill, Platt, & Jack Windsor, 2008; Moharamzadeh, Brook, Scutt, Thornhill, & Van Noort, 2008; Moharamzadeh, Franklin, Brook, & van Noort, 2009). Mice immunized with ovalbumin (OVA) in combination with HEMA have higher levels of immunoglobulin G (IgG) and IgE anti‐OVA antibodies in blood than mice immunized with OVA without any methacrylate monomer (Sandberg, Kahu et al., 2005).

Although there have been several studies demonstrating the effects of HEMA and TEGDMA on the immune system, very little is known about the immunomodulatory properties of EMA and DEGDA.

In the present study, we hypothesize that EMA and DEGDA, similar to HEMA and TEGDMA, have the ability to interfere with different immune responses. The aim of the present study was to investigate how the methacrylate/acrylate monomers EMA and DEGDA affect the immune system in vivo and in vitro, in comparison to HEMA and TEGDMA.

## 2 MATERIALS AND METHODS

### 2.1 Exposure of mononuclear cells from human blood to methacrylate/acrylate monomers

Peripheral blood mononuclear cells (PBMCs) from healthy blood donors (*n* = 8) were obtained from Sahlgrenska University Hospital. PBMCs from each donor were assayed separately and isolated by density gradient centrifugation using Ficoll‐Paque Plus (GE Healthcare Bio‐Sciences, Uppsala, Sweden). The cells were resuspended in Dulbecco's Modified Eagle's Medium (Invitrogen, Lidingö, Sweden) supplemented with 5% heat‐inactivated human AB serum (Sigma‐Aldrich, Steinheim, Germany), 100 U·ml^−1^ of penicillin, and 100 μg·ml^−1^ of streptomycin (Invitrogen). Cell viability was determined by staining with 0.4% trypan blue (Sigma‐Aldrich), and the cells were counted using a Bürker chamber.

PBMCs (2 × 10^6^ cells/per well) were cultured with or without 500 or 1,000 μM HEMA, TEGDMA, EMA, or DEGDA (duplicates) in 24‐well plates and cultured at 37 °C (humidified atmosphere, 5% CO_2_) for 24 hr. Cells that were exposed to HEMA, TEGDMA, or EMA had viability levels in the range of 90–95%, whereas more than 50% of the cells that were exposed to DEGDA died.

### 2.2 Cytokine measurements

The 21plex Group II and 27plex Group I cytokine panels (Bio‐Plex Pro™ Human Cytokine Assay; Bio‐Rad Laboratories, Hemel Hempstead, UK) were used to measure the cytokines, chemokines, and growth factor levels in the culture supernatants according to the manufactures instructions. In brief, supernatants were incubated with color‐coded beads that were conjugated to antibodies directed against specific cytokines for 1 hr. A biotinylated detection antibody was added and allowed to bind for 30 min, and thereafter, the samples were incubated with streptavidin–phycoerythrin for 10 min. A washing series was performed after each step to remove unbound protein. The concentrations of the cytokines were measured using the Bio‐Plex 200 instrument equipped with the BioManager analysis software (BioRad Laboratories), and the measured fluorescence intensities were compared to a standard curve.

### 2.3 Animals

Female, 6‐week‐old BALB/c mice (Charles River Laboratories, Sulzfeld, Germany) were used throughout the study and were kept in the animal facility according to governmental rules. The Ethical Committee for Animal Experimentation in Gothenburg, Sweden, approved the protocols (N186/15).

### 2.4 Immunization

Animals (*n* = 8/group) were immunized with the primary dose (50 μl of the test solution, which contained OVA, OVA + HEMA, OVA + TEGDMA, OVA + EMA, or OVA + DEGDA), administered subcutaneously at the base of tail (Table 1). Three weeks later, the animals were given an identical booster injection. Two weeks after the booster injection, the animals were sacrificed and splenectomized.

**Table 1:** Description of the various mouse groups and the agents that they received in vivo

	OVA 50 μg/animal	Acrylate 20 μmol/animal
Group 1	+	HEMA
Group 2	+	TEGDMA
Group 3	+	EMA
Group 4	+	DEGDA
Group 5	+	—

*Note*. OVA = ovalbumin; HEMA = hydroxyethyl methacrylate; TEGDMA = triethylene glycol dimethacrylate; EMA = ethyl methacrylate; DEGDA = diethylene glycol diacrylate.

### 2.5 Cell cultures

Spleens were removed from the mice and passed through a cell strainer (Falcon, Bergman Labora, Upplands Vasby, Sweden). The cell suspension from each spleen was assayed separately, washed, and centrifuged with ice‐cold Dulbecco's phosphate buffered saline (PBS) that lacked Ca and Mg ions (Invitrogen). The cell pellet was resuspended in PBS and isolated by centrifugation using Ficoll‐Paque Plus. The cells were washed with PBS, centrifuged, and resuspended in Iscove's Modified Dulbecco's Medium (Sigma‐Aldrich) that was supplemented with 100 U·ml^−^^1^ of penicillin, 100 μg·ml^−1^ of streptomycin, and 5% fetal bovine serum (Invitrogen).

The cells were counted, and the viability was determined by staining with 0.4% trypan blue. Cells were seeded at 2 × 10^5^ per well in 96‐well plates and stimulated with 40 μg·ml^−1^ OVA for 3 days at 37 °C (humidified atmosphere, 5% CO_2_) in triplicates. The supernatants were frozen until cytokine analysis. All the mice in Group 4 died after the booster injection.

### 2.6 Cytokine production in spleen cell cultures

The levels of IL‐1β, IL‐2, IL‐4, IL‐6, TNF‐α, and the keratinocyte‐derived chemokine (KC) that is functional homologues of IL‐8 in the splenocyte culture supernatants were measured using a commercially available cytokine kit (Bio‐Plex Pro™ mouse Cytokine Assay; Bio‐Rad Laboratories), in accordance with manufacturer's instructions. The concentrations of the cytokines were measured using the BioPlex 200 instrument equipped with BioManager analysis software (BioRad Laboratories).

### 2.7 IgG anti‐OVA antibody levels

The levels of IgG anti‐OVA antibodies in mouse sera were measured by coating plates (Maxisorp Immuno plate; Nunc, Kamstrup, Denmark) overnight at 4 °C with OVA (10 μg·ml^−1^) dissolved in PBS. The following day, the plates were washed with PBS supplemented with 0.05% Tween 20 (Sigma‐Aldrich), blocked with PBS‐0.05% Tween‐0.1% Bovine serum albumin (BSA) and washed again before incubation with diluted (1:1,000) serum for 2 hr. The plates were washed before Alkaline phosphatase (ALP)‐labeled rabbit anti‐mouse IgG antibody (Mabtech, Nacka Strand, Sweden) was added and washed again before adding 4‐nitrophenyl phosphate disodium salt hexahydrate (Sigma‐Aldrich) at 1 mg·ml^−^^1^ for 1 hr. The resulting color intensity was read spectrophotometrically at 405 nm.

A standard control and positive control, comprised of a pool of serum samples taken from all mice in Group 5, were included on each plate.

### 2.8 Hierarchical Clustering Explorer

The Hierarchical Clustering Explorer software (University of Maryland, College Park, MD, USA) was used to create a heat map for the expression of selected cytokines produced by human PBMCs exposed to methacrylate/acrylate monomers.

### 2.9 Statistical analysis

The GraphPad Prism Software (GraphPad Software Inc., San Diego, CA, USA) was used to create the artwork and analyses. For all tests, a *p* value <.05 was considered statistically significant. Statistical comparisons between paired samples were made using the Wilcoxon matched‐pairs signed‐rank test. For unpaired samples, the Mann–Whitney *U* test was used.

## 3 RESULTS

### 3.1 Cytokine production in vitro by PBMCs exposed to methacrylates/acrylates

**Figure 1:**
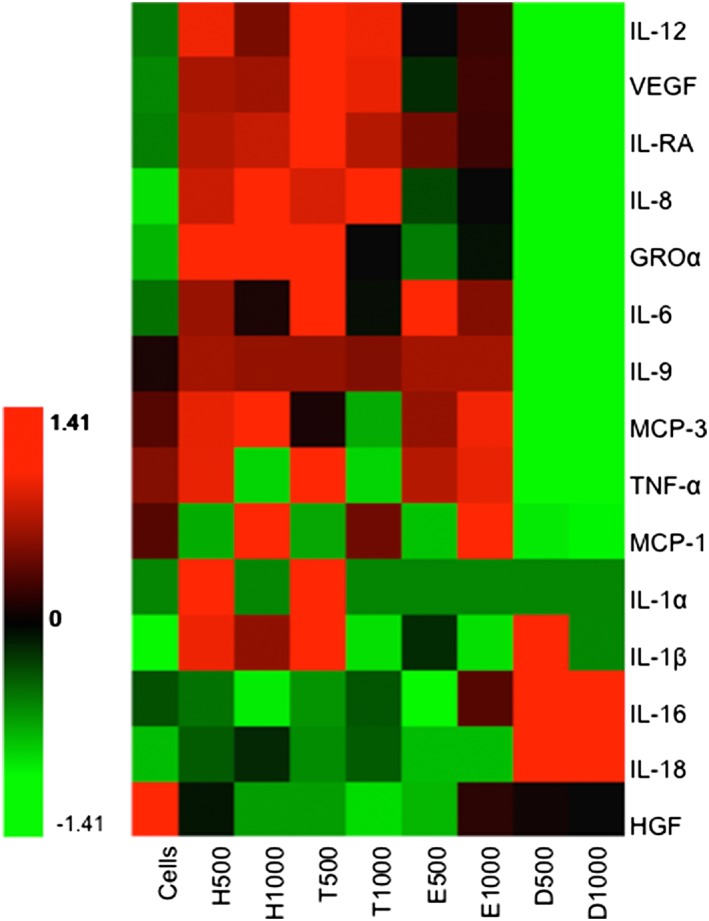
Human peripheral blood mononuclear cells (*n* = 8) were exposed in vitro to two different concentrations (500 and 1,000 μM) of hydroxyethyl methacrylate (H), triethylene glycol dimethacrylate (T), ethyl methacrylate (E), or diethylene glycol diacrylate (D). The levels of cytokines interleukin (IL)‐1β, IL‐1A, IL‐1Rα, IL‐6, IL‐8, IL‐9, IL‐12, IL‐16, IL‐18, vascular endothelial growth factor (VEGF), Growth‐Regulated Alpha Protein, monocyte chemotactic protein (MCP)‐1, MCP‐3, hepatocyte growth factor (HGF), and tumor necrosis factor (TNF)‐α in the culture supernatants were measured with a multiplexed bead‐based cytokine immunoassay. The median level for each cytokine was calculated, and the values were normalized and transformed into a heat map using Hierarchical Clustering Explorer and color codes that depicted higher (red), intermediate (black), and lower (green) expression of each cytokine

**Figure 2:**
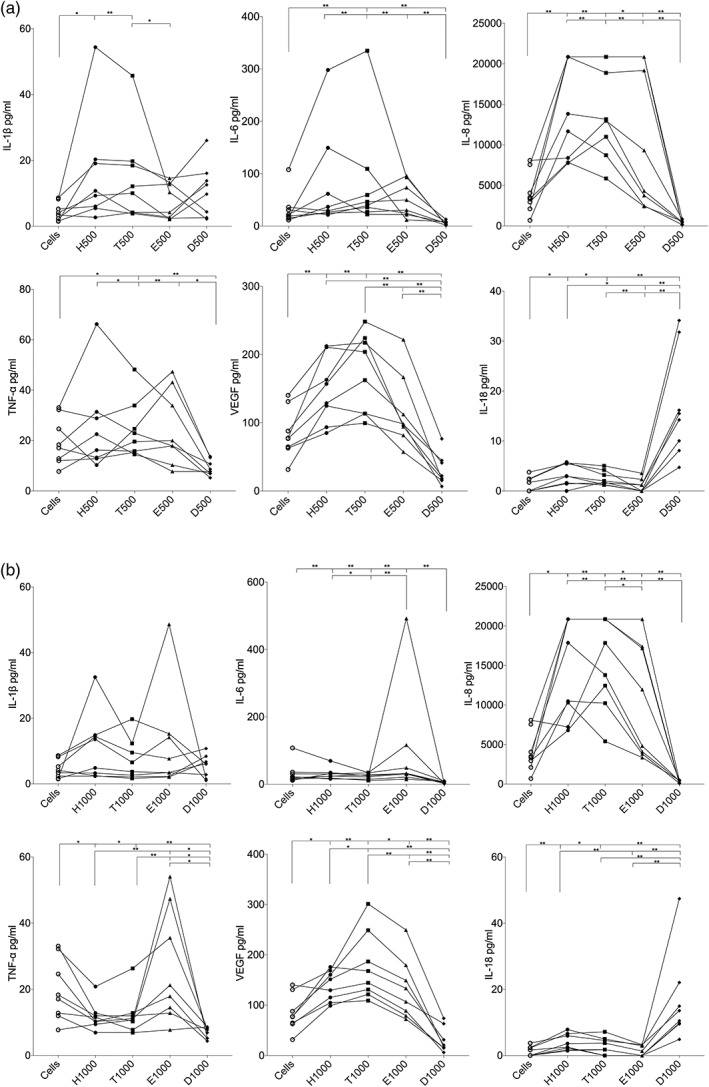
(a) Human peripheral blood mononuclear cells (*n* = 8) were exposed in vitro to 500 μM of hydroxyethyl methacrylate (H), triethylene glycol dimethacrylate (T), ethyl methacrylate (E), and diethylene glycol diacrylate (D). The levels of the cytokines interleukin (IL)‐1β, IL‐6, IL‐8, tumor necrosis factor (TNF)‐a, IL‐18, and vascular endothelial growth factor (VEGF) in the culture supernatants were measured using a multiplexed bead‐based cytokine immunoassay. Statistical comparisons were performed with the Wilcoxon matched‐pairs signed‐rank test; *p < .05; **p < .01. (b) Human peripheral blood mononuclear cells (*n* = 8) were exposed to 1,000 μM of H, T, E, and D in vitro. The levels of the cytokines IL‐1β, IL‐6, IL‐8, TNF‐α, IL‐18, and VEGF in the culture supernatants were measured using a multiplexed bead‐based cytokine immunoassay. Statistical comparisons were performed with the Wilcoxon matched‐pairs signed‐rank test; *p < .05; **p < .01

Cultures of human PBMCs (*n* = 8) were exposed for 24 hr to the methacrylate/acrylate monomers HEMA, EMA, TEGDMA, and DEGDA (at 500 and 1,000 μM). The cytokine levels in the culture supernatants were measured using the 21plex Group II and 27plex Group cytokine arrays. All the cytokines produced from cultures exposed to the monomers that had median levels >10 pg·ml^−1^ were included in a heat map (Figure 2). The cytokine expression patterns were similar in the cultures exposed to 500 μM TEGDMA and 500 μM HEMA. However, DEGDA only caused an increase in the production of IL‐1β, IL‐16, and IL‐18 (Figure 1). Cells that were exposed to EMA did not show any substantial cytokine production in vitro.

To study the differences in the immunological response between the control cells and cells that were exposed to different methacrylate/acrylate monomers, the expression levels of six typical pro‐inflammatory cytokines/chemokine (IL‐1β, IL‐6, IL‐8, IL‐18, TNF‐α) and one cytokine important for angiogenesis, that is, vascular endothelial growth factor (VEGF), produced by the PBMCs in response to each methacrylate/acrylate concentration were selected for statistical analysis (Figure 2a,b). The production of IL‐1β, IL‐8, and IL‐18 was significantly increased after exposure of PBMCs to 500 μM of HEMA or TEGDMA (Figure 2a), whereas the production of IL‐6 and TNF‐α was increased only after TEGDMA exposure. The production of VEGF was also significantly increased after exposure to HEMA or TEGDMA, as compared to the control cells. EMA exposure resulted in significantly increased production of IL‐8, whereas DEGDA exposure resulted in a significantly increased production of IL‐18 and a significantly decreased production of IL‐6, IL‐8, VEGF, and TNF‐α.

**Figure 3:**
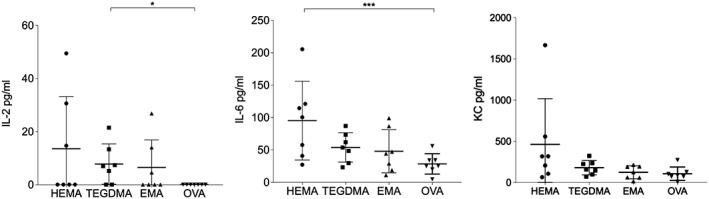
BALB/c mice (*n* = 8/group) were immunized subcutaneously at the base of the tail with 50 μl containing 50 μg/mouse ovalbumin (OVA), either alone or in combination with 20 μmol/mouse hydroxyethyl methacrylate (HEMA), triethylene glycol dimethacrylate (TEGDMA), ethyl methacrylate (EMA), or diethylene glycol diacrylate. An identical booster injection was given 3 weeks after the first immunization. All the mice in the group that received diethylene glycol diacrylate in combination with OVA died after the booster immunization. Two weeks after the booster injection, the animals were sacrificed and splenectomized. The splenocytes from seven mice/group were re‐exposed to OVA in vitro for 3 days, and the concentrations of interleukin (IL)‐2, IL‐6, and keratinocyte‐derived chemokine (KC) were determined in the culture supernatants using a multiplexed bead‐based cytokine immunoassay. Each dot represents the results from splenocytes from one individual mouse. Statistical comparisons were performed using the Mann–Whitney test; *p < .05; **p < .01; ***p < .005

The concentration of IL‐8 and VEGF in the PBMC culture supernatants was significantly increased after exposure to 1,000 μM of HEMA, TEGDMA, or EMA, as compared to the control cells (Figure 2b). The concentration IL‐18 was significantly increased after exposure to 1,000 μM HEMA or TEGDMA (Figure 3b). Overall, HEMA and TEGDMA exposure resulted in the highest cytokine production.

### 3.2 Cytokine production by spleen cells in vitro

Cytokine production was measured in the supernatants of cultures of splenocytes obtained from mice that were immunized with OVA, OVA + HEMA, OVA + TEGDMA, or OVA + EMA. OVA (40 μg·ml) was added to the in vitro cultures and were incubated for 3 days, after which the culture supernatants were collected and the levels of cytokines (IL‐1β, IL‐2, IL‐4, IL‐6, KC, and TNF‐α) were measured. There was no in vitro production of IL‐1ß, IL‐4, or TNF‐α. However, there was a significantly increased OVA stimulated production of IL‐2 in the splenocyte cultures prepared from spleen of mice that were immunized with OVA in combination with TEGDMA compared to mice immunized with OVA alone. IL‐6 production was significantly increased in the supernatants of the cultures of splenocytes obtained from mice that were immunized with OVA in combination with HEMA (Figure 3).

### 3.3 IgG anti‐OVA antibody levels

Mice (*n* = 8/group) were immunized subcutaneously at the base of the tail with OVA alone or in combination with HEMA, TEGDMA, EMA, or DEGDA, and the levels of the serum IgG anti‐OVA antibodies were measured using ELISA.

**Figure 4:**
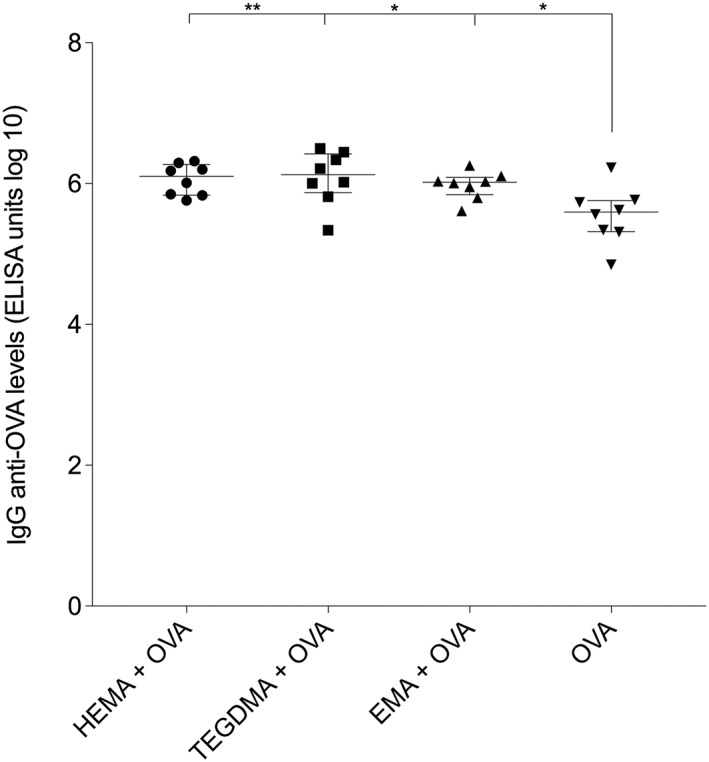
Effects of immunization of mice with ovalbumin (OVA) in combination with hydroxyethyl methacrylate (HEMA), triethylene glycol dimethacrylate (TEGDMA), or ethyl methacrylate (EMA) on the immunoglobulin G (IgG) anti‐OVA antibody levels in blood. Serum samples were collected from the animals described in Figure 3. The levels of IgG anti‐OVA levels in the sera were analyzed with an ELISA. Statistical comparisons were performed using the Mann–Whitney test; *p < .05; **p < .01

The animals immunized with OVA together with HEMA, TEGDMA, or EMA had significantly higher serum IgG anti‐OVA antibody activities compared to the mice immunized with OVA alone. There were no significant differences between the different groups immunized with OVA in combination with any of the methacrylates (Figure 4). All the animals that were immunized with OVA in combination with DEGDA died.

## 4 DISCUSSION

Dental methacrylates/acrylates are commonly used in resin‐based dental restorative materials, as well as in dental‐bonding agents. Due to incomplete polymerization or resin degradation, the monomers are released into the oral cavity (Geurtsen, 2000). Previous studies have shown that this release is time‐dependent, with approximately 90% of the unreacted monomers being released during the first 24 hr postpolymerization (Ferracane, 1994).

Monomers released from the fillings into the oral cavity may cause local and systemic effects, and they may also diffuse into the pulp via the dentin tubuli, resulting in pulpal inflammation (Geurtsen, 2000; Nicholson & Czarnecka, 2008; Schmalz, Krifka, & Schweikl, 2011).

Inflammation is a process that is regulated by cytokines, which are specialized signaling molecules. When foreign compounds, such as methacrylate/acrylate monomers, penetrate the mucosal epithelium of the mouth or the dentin, they may interact with different cells of the immune system (Reichl et al., 2002). Affected cells produce various cytokines with different effects on the surrounding tissues. Some of the produced cytokines trigger inflammation, whereas others act to limit the inflammatory lesion. Among the cytokines that promote inflammation are IL‐1, IL‐6, IL‐8, and TNF‐α. Inflammation can be suppressed by anti‐inflammatory cytokines, such as IL‐1Rα and IL‐10. Furthermore, there are cytokines that regulate angiogenesis and promote wound healing, for example, VEGF. Many of these cytokines are produced by macrophages (Janeway & Medzhitov, 2002).

Previous studies have shown increased production of the pro‐inflammatory cytokines IL‐6 and IL‐8 from dental pulp mesenchymal stem cells following exposure to HEMA in vitro (Trubiani, Cataldi, De Angelis, D'Arcangelo, & Caputi, 2012). These are important cytokines related to inflammation (Trubiani et al., 2012). The production of IL‐6 and IL‐8 has also been demonstrated after exposure of oral epithelial cells to TEGDMA (Schmalz, Schweikl, & Hiller, 2000). A study conducted by Noda et al. (2003) demonstrated suppression of TNF‐α secretion by the human THP‐1 monocyte cell line after exposure to TEGDMA and HEMA. The mainly reported effect on the immune system due to DEGDA and EMA is the occupational allergic contact dermatitis caused by them (Aalto‐Korte et al., 2007; Kiec‐Swierczynska, 1996). Many different immunomodulatory effects caused by HEMA and TEGDMA have been reported. However, there have been no previous studies investigating the immunomodulatory effects due to exposure to EMA or DEGDA, neither comparing the effects on the immune system caused by HEMA, TEGDMA, EMA, and DEGDA.

In the present study, we exposed human PBMCs to two different concentrations (500 and 1,000 μM) of four common dental methacrylates/acrylate (HEMA, TEGDMA, EMA, and DEGDA) for 24 hr. The concentrations used for the in vitro studies lie within the range of concentrations found clinically in the pulp (Noda et al., 2002). After exposure, cell viability was calculated, and 90–95% of the cells that were exposed to HEMA, TEGDMA, and EMA were viable, whereas <50% of the cells exposed to DEGDA were viable. A pattern was observed whereby most of the cytokines were present at higher levels after exposure to HEMA or TEGDMA. Because many of the cells died after exposure to DEGDA, the only observed cytokine to be significantly increased was IL‐18. This indicates that the IL‐18 was produced early after DEGDA exposure, that is, before most of the cells died. DEGDA appears to be more toxic than the other metacrylate monomers used in the present study. This is in congruent with previous studies that have suggested acrylates to be more cytotoxic compared to methacrylates (Dillingham, Lawrence, Autian, & Schmalz, 1983; Yoshii, 1997).

We have previously shown that BALB/c mice immunized with OVA in combination with HEMA produced significantly higher IgG and IgE anti‐OVA antibody levels in blood than mice immunized with OVA without any of the methacrylate monomers (Sandberg, Kahu et al., 2005). In the present study, we immunized BALB/c mice twice, 3 weeks apart, with OVA alone or in combination with HEMA, TEGDMA, EMA, or DEGDA. OVA was used to explore the adjuvant properties of the methacrylates/acrylate, because it is a common model antigen used in studies to assess adjuvant activity (Basto et al., 2015; Larsen, Lund, Thygesen, Poulsen, & Nielsen, 2003). The mice were sacrificed 2 weeks after the booster injection. Splenectomy was performed, and blood samples were obtained from all the groups, except the mice that were immunized with OVA in combination with DEGDA because all these mice died after the booster injection. This latter outcome appears to be congruent with the in vitro toxicity of DEGDA. In addition, the fact that the mice died after the booster injection indicates that DEGDA produced a very prominent memory immune response to OVA upon the primary injection. During the first exposure, it takes time for the antigen to be presented to lymphocytes and for memory cells to be produced against that antigen. During the next exposure to the same antigen, the memory cells will recognize the antigen and will initiate a faster and stronger response. A hypothesis is that memory cells are produced during the first exposure to OVA and DEGDA, and after the second immunization, a stronger response against OVA leads to death of the animals. Further studies are of interest in order to establish the exact mechanism behind this effect caused by DEGDA.

Mice that received OVA in combination with HEMA, TEGDMA, or EMA developed higher IgG anti‐OVA antibody activities than the mice that were immunized with OVA alone. We could not demonstrate any significant difference in antibody levels between the mice that received the various methacrylate monomers. However, HEMA and TEGDMA seemed to trigger higher serum IgG anti‐OVA antibody levels than the mice immunized with OVA in combination with EMA. Because EMA and TEGDMA, just as HEMA, have the capacities to act as adjuvants in vivo and to enhance the antibody response to an antigen, they may also contribute to the initiation of allergy and/or immune responses to other substances, such as bacteria and food particles, present in the oral cavity. Another interesting reflection is that the adjuvant properties of the methacrylates may be one of the causes behind the previously reported ability of them to cause allergic contact dermatitis (Kiec‐Swierczynska, 1996).

The spleen cells from the immunized mice were stimulated with OVA in vitro, and cytokine production was measured. All the groups had increased IL‐6 levels compared to the control group (mice immunized with OVA alone), although significantly increased production of IL‐6 was only observed in the group immunized with OVA in combination with HEMA. These results are in agreement with the outcome of our previous study (Andersson & Dahlgren, 2011a). The production of IL‐2, which is a reflection of OVA specific T cell proliferation in the splenocyte cultures (Boyman & Sprent, 2012), was significantly increased in the cultures from the animals that were immunized with OVA in combination with TEGDMA. No significant differences were observed for the levels of KC; however, the group that was immunized with HEMA + OVA seemed to have higher level of KC secretion than the other groups.

In the present study, we show that cytokine secretion by immunocytes is affected by exposure to methacrylate/acrylate monomers in vitro. The methacrylate monomers act as adjuvants, resulting in increased antibody production and T cell activity in mice immunized with OVA combined with a methacrylate.

The different methacrylate/acrylate monomers did not present a uniform response pattern by the exposed cells. Instead, the different monomers modulated cytokine production, inducing both increases and decreases of different cytokines. A significant finding was that the acrylate monomer DEGDA has substantially higher toxicity/inflammatogenic properties than the other methacrylate monomers.

## CONFLICT OF INTEREST

The authors declare no conflicts of interest.
